# Hæmatology—VII

**Published:** 1910-07-09

**Authors:** 


### Pathology.

### HÆMATOLOGY—VII.

For beginners, staining blood-films with hæmatoxylin and eosin is the most satisfactory for the study of the leucocytes; later, after the initial points have been mastered, Leishman's stain may be employed, as it brings out other points, such as the different granules, mast-cells, etc. As a standard or basis, five types of leucocytes may be described as occurring in normal blood, these being as follows:—

I. *The Lymphocyte* (or *Small Lymphocyte*).—These are small cells about the same size as a red-blood corpuscle, measuring on an average 7 to 8 $\mu$ in diameter. A large nucleus staining a deep purple or blue colour practically fills the entire cell, a very thin rim of protoplasm encirling this. The latter stains faintly and is usually free of granules, though at times one or two deep purple-staining granules may be present. In fresh blood two little black specks or dots are often seen in the protoplasm round the nucleus; these must not be mistaken for malarial pigment, which is never seen in the lymphocytes. They form 15 to 30 per cent. of the leucocytes of normal blood.

II. *The Large Mononuclear Cell* (synonyms, *the Large Lymphocyte, Hyaline Cell, Large Mononuclear Clear Cell*).—The typical size of this type of cell is 12 to 15 in diameter—that is, almost double the size of the small lymphocyte. In addition there are other easy points of distinction: the protoplasm present is abundant and hyaline, the nucleus, usually eccentrically placed, only fills a small portion of the cell and further stains less deeply. Stained by hæmatoxylin and eosin the protoplasm generally comes out a bluish colour, but is quite clear (hyaline) and devoid of granules; with modifications of Romanowsky's stain different granules are from time to time seen. This leucocyte is phagocytic and slightly amœboid, and in malarial blood the characteristic pigment (melanin) may be seen in such cells either when stained or in the fresh condition, the fact of their being unusually numerous in this class of blood rendering them more easily seen. In normal bloods the percentage is very small, 2 to 3 per cent. or lower.

In many bloods there is no difficulty in saying at once which type of cell I. or II. is present, no intermediate forms existing, but in other bloods, as Cabot specially points out, one must not draw too marked a line, as in many cases every intermediate form may be found between the two, and then all one can say is which size in general predominates. The majority of bloods do not have this double type.

III. *The Transitional Leucocyte of Ehrlich*.—The transitional leucocyte is about the same size as the large mononuclear cell just described. It differs in having an indentation in its nucleus, which may be very slight or, on the other hand, may be so advanced as almost to divide the nucleus into two, giving the structure the appearance of a horse's shoe. The nucleus stains somewhat faintly, and the protoplasm is generally hyaline, though at other times fine granules may be seen in it. They are supposed by some to be large mononuclear leucocytes changing into the next class, the polymorphonuclear, and some even do not give them a special name, simply including them in the large mononuclear class. As they are so characteristic it does no harm to call them transitional leucocytes, and they can afterwards be easily included in any other group.

IV. *The Polymorphonuclear Leucocyte* (synonyms, the *Polynuclear Leucocyte*, the *Finely Granular Oxyphile Leucocyte*).—These are the common leucocytes (the phagocytes) of the blood. The nucleus is not multiple really, it is all in one piece, but the shapes of individual ones are very variable. It stains deeply with hæmatoxylin and other stains. Fine granules, which have an affinity for eosin or other acid stains (oxyphile), are found in the protoplasm, and this may also contain foreign matters, such as malarial pigment or pieces of grit and dust. This merely indicates the phagocytic power of the cells, which are actively amœboid. They form 60 to 70 per cent. of the total leucocytes.

V. *The Eosinophile Leucocyte* (synonym, the *Coarsely Granular Oxyphile*).—The eosinophile leucocyte also has a polymorphous nucleus, which often only has two pieces to it and stains less deeply than that of the polymorphonuclear. The main characteristic of the cell is the presence of large highly refractile granules which have a strong affinity for eosin or other acid stains (the eosinophile or coarsely granular oxyphile granules). The granules are spherical or oval. The cell is also actively amœboid; they are scanty in normal blood, forming 2 to 4 per cent. of the total.

VI. *The Mast Cell*.—In the mast cell the nucleus usually consists of three parts, but the granules are so large and numerous as often to obscure entirely this structure. They (the granules) have a strong affinity for basic stains (methylene blue), and they are well brought out by Leishman's stain, borax methylene blue, or common methylene blue. Hæmatoxylin does not demonstrate them at all, and therefore should not be used. Mast cells are commonly found in connective and other tissues; they are found only in abnormal conditions.

VII. *Myelocytes* or *Marrow Cells*.—The myelocytes are one of the normal forms of cell found in the bone marrow. They are large cells, 12 to 20 in diameter, have a single nucleus often somewhat eccentrically placed, an abundant protoplasm and different forms of granules in this. Three types are described: (*a*) with neutrophilic granules; (*b*) with fine eosinophile granules; (*c*) with coarse eosinophile granules. These latter two are, of course, at once told from the ordinary eosinophile cell by their size and by the fact of their having a single (not a polymorphous) nucleus. Myelocytes are found in large numbers in leukemia, in small numbers in pernicious anæmia and some other obscure conditions.

